# How Do Non-Agricultural Employment and Regional Selection Affect Farmers’ Domestic Sewage Discharge Behavior? Empirical Evidence from Rural China

**DOI:** 10.3390/ijerph191710694

**Published:** 2022-08-27

**Authors:** Haiqin Ju, Jia Chen, Jingwen Xu, Hongxiao Zhang

**Affiliations:** 1 Department of Agriculture and Forestry Economics, School of Economics and Management, Nanjing Forestry University, Nanjing 210037, China; 2 Department of International Trade, School of Finance and Economics, Wuxi Institute of Technology, Wuxi 214121, China

**Keywords:** non-agricultural employment, regional selection, domestic sewage discharge behavior, environmental cognition

## Abstract

In many countries, along with rising rural labor transfer, the problem of rural domestic sewage discharge is becoming increasingly serious due to labor shortages in the villages. It is urgent to solve the environment pollution and health problems of residents which is caused by the massive discharge of domestic sewage in rural areas. Based on the survey data collected from Nanjing Agricultural University in 2020, this paper employs the ordered probit model and the CMP method, to empirically test the impact of non-agricultural employment and regional choice on farmers’ domestic sewage discharge behavior and the moderating effect of environmental cognition and the social network. The results show that: (1) There is a significantly positive correlation between non-agricultural employment and farmers’ sewage treatment behavior. (2) Environmental cognition significantly improves the participation of urban non-agricultural employment farmers in sewage treatment, and the social network has a significant role in promoting the adoption of sewage treatment behavior of local non-agricultural employment farmers. (3) Further heterogeneity analysis results show that the inhibitory effect of urban non-agricultural employment on random sewage discharge is more pronounced than that of local non-agricultural employment. Therefore, in order to effectively solve the problem of rural domestic sewage discharge, it is necessary to actively guide the sewage discharge behavior of non-agricultural employment households, strengthen the social network interaction within the village, and increase the publicity for sewage discharge knowledge.

## 1. Introduction

The results of the second national pollution source census in 2017 showed that the emission of rural water pollutants reached 5.9226 million tons in China, accounting for 47% of the total national water pollutants [1,2]. Among them, the random discharge of domestic sewage by farmers is one of the main sources of rural water pollution. Therefore, the Chinese government has carried out a series of actions to improve the rural living environment, focusing on the treatment of domestic sewage directly discharged by farmers. The rural water pollution has been mitigated under the strong impetus of government-led governance measures. However, due to the fact that rural sewage discharge has the characteristics of small and large pollution sources and wide and scattered pollution areas, the role of government investment is relatively limited for the vast number of rural areas, which fail to fully achieve the expected sewage treatment policy objectives [3,4]. In fact, farmers are not only the main body of sewage discharge, but also the beneficiaries of rural environmental improvement [5]. Thereby, guiding and regulating the farmers’ sewage treatment behavior is the key point to solve the rural environmental problems.

With the continuous expansion of urbanization and industrialization in China, in order to obtain higher working remuneration and improve family livelihood, a large number of the rural labor force have been transferred into non-agricultural industries [6]. The monitoring survey report of migrant workers in 2020 shows that the number of migrant workers in China has reached 286 million. Non-agricultural employment not only indicates the conversion of occupational types of farmers, but also emphasizes working transfer in a spatial sense. In the context of non-agricultural employment, the transition of household occupational types from farmers to workers, and the transfer of work areas from rural to urban areas all have an impact on farmers’ internal psychology and external living environment, which leads to a change in the ecological behavior of farmers [3,7].

In theory, some scholars believe that non-agricultural employment can promote farmers’ participation in environmental governance through an income effect, which is mainly manifested in the improvement of environmental literacy and the enhanced investment ability in environmental governance caused by the increase of non-agricultural income [8]. In addition, Shi et al. (2011) found that non-agricultural employment can promote farmers’ sewage treatment by broadening farmers’ access to information and strengthening interpersonal communication [9]. Some scholars argue that non-agricultural employment may weaken farmers’ willingness to participate in environmental governance. With the transfer of family population to non-agricultural industries, farmers’ sense of belonging and dependence on villages are greatly reduced, resulting in lower expected benefits and higher psychological costs of sewage treatment, thus reducing farmers’ willingness to treat sewage [10].

Therefore, there have been abundant studies on the impact of non-agricultural employment on farmers’ sewage treatment behavior However, there are two aspects that need to be considered, which are to be the contributions of this paper. First, although non-agricultural employment conceptually emphasizes the professional transformation of farmers’ family members from agriculture to non-agricultural industries, it also implies in a broad sense the geographical migration of rural and urban labor [11]. Nevertheless, existing studies only focus on the influence of non-agricultural employment as a whole employment type on sewage treatment behavior, and few discuss the heterogeneous effect of different regional choices of farmers’ non-agricultural employment on sewage treatment behavior. In fact, there may be some differences in sewage discharge behavior of the non-agricultural employment farmers in different spatial ranges. This paper further considers the differential impact of the spatial heterogeneity of rural households on the relationship between non-agricultural employment and sewage treatment behavior, which is helpful for the effective implementation of sewage treatment in different populations. Second, most of the researches take the endogenous variable-non-agricultural employment, as a binary dummy variable, and adopt the probit model for the empirical test. Indeed, the effect of farmers’ non-agricultural employment on sewage treatment behavior may cause potential endogeneity problems due to missing variables or reverse causation. Hence, this paper employs the ordered probit model to gradually analyze the marginal effect of non-agricultural employment on sewage treatment behavior, and uses the conditional mixed estimation (CMP) to solve the endogeneity problem caused by reverse causation. Therefore, the survey data of farmers in Jiangsu Province in 2020, and the ordered probit model are used here to explore the impact of non-agricultural employment on the domestic sewage discharge behavior of farmers. At the same time, through the division of different types of non-agricultural employment, the internal impact mechanism of local non-agricultural employment and urban non-agricultural employment on sewage discharge behavior is investigated.

The remainder of the paper is organized as follow. Section 2 provides the theoretical analysis, Section 3 introduces the data with the variable and estimation strategy. Econometric evidence is presented in Section 4, and Section 5 summarizes the conclusions.

## 2. Theoretical Analysis

Non-agricultural employment plays an important role in strengthening farmers’ family endowments [12,13]. In terms of domestic sewage treatment, this strengthening effect may influence the farmers’ behavior by the following two aspects. On the one hand, capital constraints will directly affect farmers’ willingness and ability to pay for domestic sewage treatment [14]. In other words, sufficient capital investment is a prerequisite for farmers to carry out environmental governance. The fundamental reason for farmers’ non-agricultural transfer is that the expected income of non-agricultural employment is greater than that of agricultural production [5]. Therefore, farmers’ non-agricultural employment can strengthen their capital endowment by increasing farmers’ household income, thereby increasing capital investment in environmental governance and promoting their domestic sewage treatment. On the other hand, non-agricultural employment can also strengthen the human capital endowment of rural households and promote household sewage treatment. Specifically, farmers can be exposed to advanced environmental protection concepts and acquire much more environmental protection knowledge in the experience of non-agricultural employment, which strengthen their cognitive ability to treat domestic sewage, and induces a positive impact on farmers’ environmental governance behavior [15].

Further, non-agricultural employment can be divided into local non-agricultural employment and urban non-agricultural employment according to the working distance of migrant workers. The distance of migrant households may affect their behavior decisions from different angles. Generally speaking, if the expected income of local employment is closed to urban non-agricultural employment, farmers are more inclined to engage in local non-agricultural employment with the consideration of part-time farming or family care. Hence, farmers choosing urban non-agricultural employment is mainly ascribed to the higher labor remuneration in economically developed cities. In other words, there is a strong correlation between farmers’ choice of urban non-agricultural employment and the economic development level of the living areas [16]. Farmers in relatively developed cities can be exposed to more concepts of environmental protection in their working life, thus helping to develop a good habit of sewage discharge [17]. At the same time, compared with rural areas, the relatively clean environment in cities can also stimulate farmers’ desire and demand to improve their hometown’s living environment, so it can strengthen the demand of migrant farmers for the improvement of the living environment, and farmers are more willing to participate in the village’s sewage treatment [18].

As analyzed above, when the expected income of local non-agricultural employment is lower than that of urban non-agricultural employment, the reasons why farmers still choose local non-agricultural employment may be for the consideration of part-time farming or family care. At this time, the production and life of local non-agricultural employment households are still embedded in the rural traditional acquaintance society. In the traditional acquaintance society, farmers always exchange life production information and make behavioral decisions based on the social relationship network formed by blood, kinship, and geography [19]. First of all, the random discharge of domestic sewage has a certain negative externality. Under the constraints of reputation loss in the acquaintance society, local non-agricultural employment farmers need to consider the possible negative impact of random dumping of sewage on surrounding neighbors. Second, the initial investment of sewage treatment equipment is relatively large and has the typical nature of public goods. Farmers generally prefer “free ride” to reduce the cost of sewage treatment. Thus, in reality, sewage treatment generally needs to be carried out in the form of collective action of the whole village. In the acquaintance social network, farmers involved in local non-agricultural employment are influenced by village ethics, social norms, and collective identity, and they will still choose to participate in the supply of public goods [20]. Meanwhile, the social network of acquaintances also helps to expand the channels of information exchange among farmers and reduce the cost of communication and negotiation [21].

Accordingly, Hypotheses 1 and 2 are proposed:

**Hypothesis** **1.** *Households with non-agricultural employment are more willing to treat domestic sewage*.

**Hypothesis** **2.** *For farmers involved in local non-agricultural employment, environmental cognition positively regulates their domestic sewage treatment behavior. For farmers involved in urban non-agricultural employment, the social network positively moderates their domestic sewage treatment behavior*.

## 3. Case Study Presentation and Methodology

### 3.1. Case Study Presentation

Jiangsu Province is located on the lower reaches of the Yangtze River and Huaihe River basins (the developed coastal areas of China mainland). It owns rich water resources and a high urbanization rate. By 2018, the province’s rural population reached 47.75 million, accounting for 59.5% of the province’s total population. Due to the large rural population base and the high per capita sewage discharge in the province, the domestic sewage discharge problem is more prominent in densely populated areas. In order to promote rural sewage treatment comprehensively, the Jiangsu provincial government has issued the “Jiangsu Province Groundwater Pollution Prevention and Control Implementation Plan”, “Jiangsu Province’s 14th Five-Year Plan for Soil, Groundwater and Rural Ecological Environmental Protection”, and “the provincial ecological environment special law enforcement action plan in 2022” and other related policy documents. With the continuous promotion of sewage treatment, the rate of rural domestic sewage treatment in the province reached 31.1% in 2022, but it still needs to be further strengthened.

### 3.2. Methodology

#### 3.2.1. Data

The data sources used for analysis are the China Land Economic Survey (CLES) conducted by the Department of Humanities and Social Sciences of Nanjing Agricultural University and the Jin Shanbao Institute of Modern Agricultural in Jiangsu Province in 2020. The reasons for selecting Jiangsu Province as the sample province in this paper are as follows. First, Jiangsu Province is located in a hilly area with abundant water sources. The per capita domestic sewage discharge in rural areas is relatively high and the problem of domestic sewage discharge is more prominent [3]. Second, Jiangsu Province is one of the main provinces of labor outflow, with a high degree of family non-agriculturalization. It is thus representative to select the farmers in this area as the research object. The questionnaire content of CLES includes the basic situation of farmers’ families, employment, the ecological environment, and other aspects, covering the key indicators needed in this paper, and providing data support for the empirical research of this paper. The sampling method of probability proportional to size (PPS) was adopted in the survey. Twenty-six counties were randomly selected from thirteen prefecture-level cities of Jiangsu Province. Two sample townships were randomly chosen from each county, and one administrative village was randomly selected from each township. Finally, the researchers randomly selected fifty households in every village. The sample included 52 villages and 2628 households. After processing the abnormal and missing values of variables, 2473 valid samples were finally retained.

#### 3.2.2. Variable Selection and Statistical Description

The explained variable in this paper is the household sewage discharge behavior. The indicator was measured by asking “How is your domestic sewage discharged”. If farmers choose to discharge it to outdoor or open ditches at will, a value of 0 is assigned. The option to discharge to the sewer is assigned a value of 1. Using a special sewage collection bucket to collect is assigned a value of 2. According to the environmental protection level of sewage discharge from low to high, the sewage discharge behavior of farmers can be divided into three categories and assigned a value of 0–2: First, “random discharge to outdoor and open ditches” = 0. The randomly scattered discharge method reflects the low participation of farmers in sewage treatment, which is not conducive to the improvement of sewage treatment effectiveness. Second, “discharge to sewer” = 1. Farmers gradually participate in the process of sewage treatment through the installation and use of water pipes, thereby curbing the pollution caused by random discharge. Third, “use a dedicated sewage collection bucket to collect” = 2. Compared with discharging into the sewer, this behavior can better reflect farmers’ stronger awareness of environmental protection and higher participation in sewage treatment. In a practical sense, it is helpful to have the assignment of three types of variables to explain the participation of farmers in sewage treatment, and improve the effectiveness of sewage treatment.

Household non-agricultural employment is selected as the core explanatory variable, which is mainly because the household is the basic unit of agricultural micro-research, and the household is generally the decision-making unit for sewage discharge. Some researchers believe that the indicator could be measured by the number of family migrants [22]. On this basis, this paper further draws on the previous research [23] and uses the non-agricultural employment ratio of family members to represent the non-agricultural employment situation of rural households.

According to the theoretical analysis, the social network and environmental cognition will positively regulate the sewage treatment behavior of farmers involved in non-agricultural employment. Drawing on the results of previous research [24], this paper selects the expenditure of human gifts as the proxy variable of social network. According to the expenditure on human gifts, it can not only accurately determine the scale of the family’s social relationship network, but also objectively identify the size of the social resources based on these networks. In addition, the questionnaire of CLES sets up the question “Is the improvement of living environment important for the improvement of the village environment”, which is used as the proxy variable of farmers’ environmental cognition level.

Considering that other factors can also interfere with farmers’ sewage discharge behavior, this paper draws on the previous research results and controls factors such as farmers’ personal characteristics, family characteristics, environmental cognition, and policy understanding. The descriptive statistics of variables are shown in Table 1.

#### 3.2.3. Model Specification and Estimation Methods

##### The Benchmark Regression Model: Ordered Probit Model

This paper uses Stata15.0 for empirical analysis. Since the explained variable in this paper is ordered and discrete, the ordered probit model is selected to analyze the influencing factors of farmers’ domestic sewage discharge behavior. The model is developed as follows:

(1) $$y^{*}\;=\;\alpha_{0}\;+\;\alpha_{1}x_{i}\;+\;\alpha_{2}c_{i}\;+\;{ℰ}_{i}\;$$

(2) $$y=\{\begin{array}{c} 0,\;\;y^{*}\in \left(-\infty ,\;r_{0}\right)\\ 1,\;y^{*}\in [r_{0,}\;r_{1})\\ 2,\;\;y^{*}\in [r_{1},+\infty ) \end{array}\;$$

where farmers’ sewage discharge behavior is denoted by *y*, and the hidden variable of farmers’ sewage discharge behavior is denoted by y*; *x_i_* represents the core explanatory variable that is the family non-agricultural employment situation, *c_i_* represents the control variables, *α_i_* (*i* = 0, 1, 2) is the column matrix of the parameters to be estimated, *ε_i_* is the random error term, and *r*_0_ and *r*_1_ are the unknown tangent points. When *ε_i_* ~ N (0, 1) is distributed, the ordered probit model can be obtained:

$$p\left(y\;=\;0\right)\;=\;p\left(y^{*}\;<\;r_{0}\right)\;=\;\Phi \left(r_{0}\;-\;\alpha_{1}x_{i}\;-\;\alpha_{2}c_{i}\right)$$

$$p\left(y=1\right)=p\left(r_{0}\;\leq \;y^{*}<r_{1}\right)=\;\Phi \left(r_{1}-\alpha_{1}x_{i}-\alpha_{2}c_{i}\right)-\Phi \left(r_{0}-\alpha_{1}x_{i}-\alpha_{2}c_{i}\right)$$

(3) $$p\left(y=2\right)=p\left(y^{*}\;\geq \;r_{1}\right)=1-\;\Phi \left(r_{1}-\alpha_{1}x_{i}-\alpha_{2}c_{i}\right)\;$$

From Equation (3), it can be found that when the household non-agricultural employment ratio is lower than *r*_0_, the household sewage is discharged to outdoor and open-air ditches at will. When it is higher than *r*_0_ and less than *r*_1_, the farmer directly discharges the domestic sewage to the sewers When it is higher than *r*_1_, farmers use special sewage collection buckets to collect domestic sewage.

##### Endogenous Discussion: CMP Method

There may be problems of missing variables and reverse causality between non-agricultural employment and sewage discharge behavior, which leads to biased empirical results. First, the omission variables that are difficult to measure, such as farmers’ subjective employment preference choices and living habits, may have an impact on non-agricultural employment and sewage discharge behavior, resulting in the problem of omission variables. In addition, when the village environment is seriously polluted by the farmers’ polluting behavior, farmers choose to go out to work for better life quality, which leads to an increase in the number of migrant workers. Therefore, the pollutant discharge behavior of households may adversely affect the family’s choice of migrant workers, which will lead to the problem of reverse causality. In order to solve the above problems, this paper uses the conditional mixed estimation (CMP) proposed by previous research [25]. In the first stage, it is necessary to introduce the instrumental variable and evaluate the correlation between instrumental variable and endogenous explanatory variable. In the second stage, in order to determine whether the core explanatory variable is endogenous according to the endogenous test parameter atanhrh_12, the instrumental variables are substituted into the model for regression. If the endogenous test parameter atanhrho_12 is significantly different from zero, it means that the estimation result of the CMP method is better than the estimation result of the ordered probit model.

##### Moderating Effect Model

Based on the above theoretical analysis, it can be seen that social network and environmental cognition may play a moderating role in the impact of non-agricultural employment on farmers’ pollutant discharge behavior. In order to test the moderating effect, this paper establishes the following moderating effect model:

(4) $$y^{*}\;=\;\beta_{0}\;+\;\beta_{1}x_{i}\;+\;\beta_{2}z_{i}\;+\;\beta_{3}(x_{i}\;\times \;z_{i})\;+\;\beta_{4}z_{i+}\mu_{i}\;$$

where the moderating variable is denoted by *z_i_*, and the interaction term is denoted by *x_i_* × *z_i_*. *β*_0_ is the constant term, *β_i_* (*i* = 1, 2) is the parameter coefficient to be estimated, and *μ_i_* is the random error term. After adding the interaction term, the moderating effect of social network and environmental cognition can be examined through *β*_3_.

## 4. Results and Discussions

### 4.1. Basic Estimate Discussions

Considering the possibility of multicollinearity among the selected variables, this paper tests the variable variance inflation factor (VIF) in the model first of all. The test results show that the VIF of all variables in this model are less than 10, which satisfies the principle of independence, so the selected explanatory variables do not have serious collinearity problems. In addition, the regression results of the model are tested by parallel lines, the chi-square value is 9.65, and the *p* value is 0.14 > 0.05, indicating that through the parallel line test, the regression equations are parallel to each other, and the ordered probit model can be used for analysis.

Table 2 shows the results of the ordered probit model. Model (1) only adds core explanatory variables. The results show that non-agricultural employment has a significant positive impact on the domestic sewage discharge behavior of farmers. Based on model (1), model (2) added some control variables, the pseudo R2 also increased from 0.004 to 0.013, indicating that the explanatory power of the model is enhanced after adding some control variables. Compared with model (2), the explanatory power of the model is further enhanced after adding all control variables to model (3). Overall, the results of model (1) to (3) are all significant, and the significance and direction of action of the explanatory variables and control variables have not changed. Therefore, it shows preliminarily that the estimated results of the impact of non-agricultural employment on farmers’ sewage discharge behavior are robust. It can be found that non-agricultural employment positively affects the sewage treatment behavior of farmers at the 1% significance level, that is, as the proportion of non-agricultural employment increases, non-agricultural employment will promote family members’ participation in sewage treatment through the enhancement of capital and human endowments. Hypothesis 1 of this paper has thus been preliminarily verified.

From the point of control variables, age, wealth level, and policy understanding have inhibited the random discharge of domestic sewage by farmers at a statistically significant level of 1%. With sufficient financial support and the guidance of environmental protection policies, families can better understand the importance of environmental protection for village development and choose to participate in the treatment of domestic sewage. Political status has a positive impact at the level of 5%, indicating that households with village cadres tend to choose a more environmentally friendly way of discharging domestic sewage, which further shows that village cadres have a better understanding of human settlement improvement policies than other villagers. The leaders of the village action, and the families with political identities, will also take the lead in governance. The proportion of women has a positive impact at the level of 10%. Families with a higher proportion of women may pay more attention to the issue of domestic sewage discharge, and the increase in the proportion of women in the family will also strengthen women’s decision-making power in family affairs, thereby promoting participation in sewage treatment. On the contrary, insignificant environmental perception means that although farmers are aware of certain environmental pollution problems in their villages, they cannot link pollution with domestic sewage discharge. Therefore, incomplete environmental perception may also be the reason for the random discharge of rural households. The fact that the average education level of households is not significant may be due to the failure to receive relevant knowledge of water resource protection in the process of school education, so that they cannot understand the importance of domestic sewage discharge behavior for the ecological environment.

### 4.2. Marginal Effects Analysis

The results of the above basic estimate can only show the impact of non-agricultural employment on sewage treatment behavior from the overall parameter sign and the significance. This paper further analyzes the marginal effect of explanatory variables. As shown in Table 3, when household members with non-agricultural employment, the corresponding coefficient of domestic sewage discharged to outdoor and open-air ditches is −0.061, which is significant at the 1% statistical level, and the coefficient sign is negative, indicating that the probability of sewage discharge to outdoor and open ditches decreased by 6.1%. Similarly, the corresponding coefficient of domestic sewage discharged to the sewers is 0.042, which is significant at the 1% statistical level, and the coefficient sign is positive, indicating that the probability of domestic sewage discharged to the sewers has increased significantly by 4.2%. Although the coefficient for collection with special sewage collection buckets is positive, the increase is not large relative to discharge to the sewer. In general, when the family members choose non-agricultural employment, the domestic sewage discharge behavior of farmers develops in a positive direction. Specifically, when the family members choose non-agricultural employment, the probability of discharging domestic sewage to the sewers will be greatly increased, while the probability of being discharged to outdoor and open ditches will be reduced. The probability of using a special sewage collection bucket to collect as the highest level of sewage discharge behavior is not as high as the probability of discharge to the sewer. The possible reasons may be that the families with a high degree of non-agricultural employment are also unable to afford the costs of domestic sewage treatment. On the other hand, the current popularity of sewage collection barrels is not high in rural areas. Although households with a high degree of non-agriculturalization have a greater advantage in information acquisition, they may not understand this sewage treatment method.

### 4.3. Endogeneity

Considering the potential endogeneity problem, this paper chooses the CMP method for further examination. The effective estimation of CMP requires the selection of a suitable instrumental variable. The “non-agricultural employment ratio of villages” as the instrumental variable is selected. On the one hand, the increase in the number of migrant workers in the village contributes to strengthen the connection between the village and the outside world and provides opportunities for other farmers to go out to work. At the same time, with the increase in the proportion of non-agricultural employment in villages, the atmosphere of non-agricultural employment will also affect the choice of other farmers to go out to work. On the other hand, the proportion of migrant workers at the village level does not directly affect the pollutant discharge behavior of rural households, so it satisfies the requirement of exogenous instrumental variables.

First, the CMP method is used to regress the ordered probit model, and the obtained regression results are basically consistent with the basic regression results. In addition, the IV-ordered probit model is estimated in two stages using the CMP method after determining the instrumental variables. From Table 4, the regression results of the first stage show that the proportion of non-agricultural employment in villages is positively correlated with the proportion of non-agricultural employment in households at the statistical level of 1%, while there is a certain correlation between instrumental variables and endogenous variables. Furthermore, the parameter of endogeneity test (atanhrho_12) is significant at the 1% statistical level, indicating that the household non-agricultural employment ratio is an endogenous explanatory variable, that is, the results of the IV-ordered probit model are better than that of the ordered probit model under the CMP method. Compared with the results of the basic estimate, the regression results of the IV-ordered probit model using the CMP method are more significant and the absolute value of the correlation coefficient is also relatively increased, which further indicates that the use of the instrumental variable can effectively solve the endogeneity problem which again verifies the reliability of the basic estimate.

### 4.4. Heterogeneity

With the above results the relevant empirical tests on the impact of non-agricultural employment on sewage discharge behavior were conducted and it was found that non-agricultural employment significantly promotes the orderly discharge behavior of farmers. However, the above results are only considered from the whole sample level, which may ignore the differences in the distance between different labors. Therefore, based on the CMP method in this paper the heterogeneity of the impact of non-agricultural employment on sewage discharge behavior under different working distances was examined in turn. The results of the heterogeneity are shown in Table 5.

According to the working distances in different regions, we divided the labor samples of non-agricultural employment into non-agricultural employment samples of local townships, non-agricultural employment samples that cross townships but not across counties, and non-agricultural employment samples that cross counties in order to analyze the effect of regional selection of employment on domestic sewage discharge behavior. The results of models (4) in Table 5 show that the non-agricultural laborers in the township are less likely to participate in domestic sewage treatment. On the one hand, considering that non-agricultural laborers in the township may have part-time jobs, when households focus on agricultural production, it may limit farmers’ access to external information, which is not conducive to the adoption of sewage treatment behavior. Models (5) and (6) in the estimated results show that the probability of non-agricultural employment across townships and non-agricultural employment is higher than that of non-agricultural employment across counties, while the influencing coefficients are not much different. The possible reason is that due to the high level of urbanization in Jiangsu Province, laborers who work across townships but not across counties could also experience the atmosphere of clean environment as felt in the city. On the other hand, laborers who work across townships but not across counties may have a stronger social network than those who work across counties due to their longer time of living in the village. The researchers believe that the improvement of the relationship network will help farmers participate in the environmental governance of the village [26]. The laborers who are employed across counties have stronger capital endowment than the laborers employed across townships but not across counties, and the ability accumulation also reaches maximum. Under the influence of the income effect, the laborers employed across the county are able to improve their domestic sewage treatment method and promote the adoption of treatment behavior.

### 4.5. Mechanism of Environmental Cognition and Social Network

Based on the models (4)–(6), further testing was conducted on the moderating role of environmental cognition and social network in the implementation of sewage treatment behavior of local non-agricultural employment farmers and urban non-agricultural employment farmers. The specific regression results are shown in Table 6. The results of model (8) show that environmental cognition promotes the adoption of urban non-agricultural employment farmers’ sewage treatment behaviors at the 5% significance level. Working in the city will help farmers improve their environmental cognition level because of the clean environment and the work life of the city. The greatly enhanced environmental cognition after returning to the village will significantly promote farmers’ participation in sewage treatment. The results of model (9) and (10) show that the social network at the 1% significance level has a promoting effect on the sewage treatment behavior of local employed farmers and urban employed farmers. The social network of acquaintances in rural areas often has a significant inhibitory effect on the random sewage discharge behavior of farmers. By comparing the interaction coefficients of model (9) and model (10), the social network has a more obvious role in promoting the sewage treatment behavior of local employed farmers. Due to the popularization of the internet and the upgrading of transportation, even if the migrant workers are far away from their hometown, it is still possible to enhance their rural social interaction through mobile phones or transportation. Therefore, it is also possible for farmers who are employed in cities or towns to implement sewage treatment because of the geographical kinship’s influence.

### 4.6. Robustness Test

Although the CMP method is used to alleviate the endogeneity problem, considering that there may still be problems such as measurement error in the regression is necessary in order to verify the reliability of the regression results. This paper adopts the following three methods to test the robustness: First, replace the core explanatory variables. “Average non-agricultural employment hours” was used to replace “the ratio of household non-agricultural employment”. Due to the instability of non-agricultural employment, farmers may have part-time jobs in reality. At this time, the interviewed farmers will have a certain degree of subjectivity due to the deviation of understanding when filling out the questionnaire. Therefore, setting the number of people as a measurement indicator in the questionnaire acquisition may cause a certain error, and the selection of the variable “Average non-agricultural employment time” is more objective, which helps to solve the above measurement error, and ensure the empirical result’s robustness. Second, change the estimation method. In addition to the ordered probit model, this paper also uses the probit model to study the impact of non-agricultural employment on the domestic sewage discharge behavior of farmers, and verify the robustness of the regression results through different estimation methods. Third, remove the sample. Based on the regression of the CMP method, this paper removes the sample of pure agricultural households, for better verification of the relationship between non-agricultural employment and farmers’ sewage discharge behavior. Table 7 reports the regression results of the robustness test.

The estimation results of the alternative explanatory variables in model (11) verify that the effect of non-agricultural employment on the domestic sewage discharge behavior of farmers is significant, but the direction is inconsistent with the basic regression’s results. The possible reason is that the increase in the non-agricultural employment time will correspondingly shorten the time of farmers in the village, which will lead to changes in the degree of attachment and sense of belonging in the village. Therefore, the longer time working outside and the lower sense of village belonging of the farmers, thereby, reduce their concern for the village environment and their participation in environmental governance. After changing the model, we found that the estimation results in model (12) also show the robustness of the basic regression. Compared with the basic regression, after removing irrelevant samples, the empirical results show as being more significant and the absolute value of the correlation coefficient increases significantly in the model (13), which further demonstrates the robustness of the empirical results.

## 5. Conclusions and Policy Implications

### 5.1. Conclusions

On the basis of theoretical analysis, based on the survey data of 2473 farmers in Jiangsu Province, China, this paper used the ordered probit model to empirically test the impact of farmers’ non-agricultural employment and regional selection on domestic sewage discharge behavior. The moderating effects of social network and environmental cognition were also tested by constructing a moderating effect model. The results demonstrate that non-agricultural employment has a significantly positive impact on the domestic sewage discharge behavior of farmers. The results of the moderating effect analysis show that environmental cognition can significantly improve the participation of urban off-farm employment households in sewage treatment, and social network can significantly promote the adoption of sewage treatment behavior of local non-agricultural employment households. Heterogeneity analysis shows that the probability of local non-agricultural employment households to implement sewage treatment behavior is lower than that of urban off-farm employment households, and the non-agricultural employment households who cross townships but not counties are more willing to implement relatively environmentally friendly sewage disposal behaviors. Our conclusions are consistent with the previous researcher [8], who also believed that non-agricultural employment would promote farmers’ participation in domestic waste management, but contrary to the other conclusions of researches [10,27], they believed that non-agricultural employment may reduce farmers’ collective action ability and sense of belonging to the village, thereby inhibiting farmers’ participation in environmental governance. These differences may be attributed to the introduction of local non-agricultural employment farmers’ survey data, which has systematically considered the impact of overall non-agricultural employment on participation in environmental governance. With the rapid development of China’s rural tertiary industry in the new era, the numbers of the local non-agricultural employment population have increased dramatically. Therefore, the different regional choices of non-agricultural employment farmers should be considered, which plays an important role in promoting the sewage discharge behavior of farmers.

### 5.2. Policy Implications

Based on the above research conclusions, this paper proposes the following policy suggestions: First, the government should guide the rural surplus labor force to engage properly in non-agricultural employment, and actively promote the local employment of farmers through the establishment of human resource platforms, expanding income channels and attracting the return of talents. While promoting non-agricultural employment, it is necessary to promote vigorously rural sewage treatment, and focus on the non-agricultural employment households with lower degree as the main promotion objects. For non-agricultural households, the government should guide and regulate their sewage discharge behaviors by providing training of environmental protection awareness and carrying out collective activities in the village to improve farmers’ adoption of sewage treatment behaviors. Second, the government should strengthen the environmental awareness level of farmers. To be more comprehensive, the government should make good use of the internet, conferences, posters, etc., to strengthen the publicity of domestic sewage discharge knowledge, thereby enhancing farmers’ awareness of sewage treatment, and strengthen the awareness of the importance of sewage treatment for village development. Third, the government should strengthen farmers’ accruing of social network. Based on the collective actions related to sewage treatment in rural areas, we can promote communication among farmers, reduce the cost for households purchasing sewage treatment equipment, and help to improve their living environment.

Due to the limitation of research resource and objective conditions, this manuscript has the following shortcomings. This article uses cross-sectional data from Jiangsu Province. Compared with the national survey data, the sample size of CLES2020 is relatively small. Since we mainly focus on domestic sewage discharge in rural areas, there are certain differences in the degree of water pollution and sewage treatment status in different regions. There may be certain limitations in exploring the impact of non-agricultural employment on sewage discharge behavior through survey data in one province. Therefore, in subsequent research, we will use national large sample data to better reveal the relationship between variables.

## Figures and Tables

**Table 1:** The definitions and descriptive statistics of the survey data.

Variable	Definition	Mean	SD
Domestic sewage discharge behavior	Discharge at will = 0, discharge to sewer = 1, collection in sewage collection bucket = 2	0.899	0.386
Household non-agricultural employment ratio	The number of the non-agricultural labor force in the household/the total number of the labor force in the household	0.412	0.291
Environmental cognition	Is the improvement of living environment important for the improvement of the village environment? (1 = very unimportant, 2 = unimportant, 3 = moderately important, 4 = somewhat important, 5 = very important)	4.203	0.993
Social network	Take the logarithm of the payment of human gifts	7.575	2.704
Female ratio	Number of women/total household size	0.484	0.159
Average family age	Average age of family members (years)	48.608	12.736
Average family education level	Average years of education of family members (years)	8.650	2.292
Political identity	Whether anyone in the family is a village cadre or party member (yes = 1, no = 0)	0.351	0.478
Wealth level	Total annual household income (ten thousand yuan)	10.784	11.412
Environmental awareness	How is the living environment in your area? (1 = no pollution, 2 = slight pollution, 3 = moderate pollution, 4 = severe pollution)	1.391	0.547
Policy understanding	Do you understand the improvement of rural living environment? (1 = haven’t heard of it, 2 = heard of it, not very sure, 3 = know a little, 4 = know, 5 = very understand)	2.818	1.240

**Table 2:** Estimated results of the impact of non-agricultural employment on farmers’ sewage discharge behavior.

Variable	(1)	(2)	(3)
Household non-agricultural employment ratio	0.290 ***	0.376 ***	0.296 ***
	(0.097)	(0.105)	(0.109)
Female ratio		0.323 *	0.342 *
		(0.184)	(0.184)
Average family age		0.006 **	0.007 ***
		(0.003)	(0.003)
Average family education level		0.004	−0.006
		(0.014)	(0.015)
Political identity		0.199 ***	0.129 **
		(0.061)	(0.062)
Wealth level			0.008 ***
			(0.002)
Environmental awareness			−0.065
			(0.054)
Policy understanding			0.079 ***
			(0.024)
Observations	2473	2473	2473
Wald chi2	8.91 ***	32.26 ***	60.90 ***
Pseudo R2	0.004	0.013	0.022

Note: ***, **, and * denote significant at 1%, 5%, and 10% level, respectively; robust standard errors are presented in parentheses.

**Table 3:** Analysis of the marginal effect: non-agricultural employment on domestic sewage discharge behavior.

Domestic Sewage Discharge Behavior	Dy/Dx	Std.Err.	Z	P > |z|
Discharge to outdoor and open ditches	−0.061	0.023	−2.71	0.007
Discharge to sewer	0.042	0.016	2.66	0.008
Use a special sewage collection bucket to collect	0.019	0.007	2.66	0.008

**Table 4:** Non-agricultural employment and domestic sewage discharge behavior of farmers: the CMP method.

Variable	Ordered Probit	IV-Ordered Probit	
		The First Stage	The Second Stage
Household non-agricultural employment ratio	0.296 ***		2.047 ***
	(0.111)		(0.377)
Village non-agricultural employment ratio		0.801 ***	
		(0.073)	
Female ratio	0.342 *	−0.095 ***	0.454 ***
	(0.176)	(0.032)	(0.168)
Average family age	0.007 ***	−0.007 ***	0.018 ***
	(0.002)	(0.000)	(0.003)
Average family education level	−0.006	0.012 ***	−0.028 **
	(0.013)	(0.002)	(0.013)
Political identity	0.129 **	0.012	0.085
	(0.064)	(0.011)	(0.062)
Wealth level	0.008 ***	0.005 ***	−0.004
	(0.003)	(0.000)	(0.004)
Environmental awareness	−0.065	0.010	0.802 *
	(0.051)	(0.009)	(0.049)
Policy understanding	0.079 ***	0.004	0.061 **
	(0.024)	(0.004)	(0.023)
Atanhrho_12		−0.507 ***	
		(0.126)	
Observations	2473	2473	
Wald chi2	55.19 ***	1181.47 ***	

Note: ***, **, and * denote significant at 1%, 5%, and 10% level, respectively; robust standard errors are presented in parentheses.

**Table 5:** Heterogeneous effects of off-farm employment on wastewater discharge behavior.

Variable	Sample of Labor Force in the Township	Samples of Labor Forces across Townships but Not across Counties	Sample of Labor Forces across Counties
	(4)	(5)	(6)
Household non-agricultural employment ratio	−0.585	4.053 ***	4.541 ***
	(1.176)	(0.552)	(0.845)
Atanhrho_12	0.201	−1.158 ***	−1.198 **
	(0.301)	(0.344)	(0.500)
**First stage estimate**			
Village non-agricultural employment ratio	0.659 ***	0.582 ***	0.336 **
	(0.123)	(0.166)	(0.149)
Control variable	YES	YES	YES
Observations	687	340	491
Wald chi2	84.57 ***	307.08 ***	229.68 ***

Note: *** and ** denote significant at 1% and 5% level, respectively; robust standard errors are presented in parentheses; limited by space, the regression results of the control variables are not reported.

**Table 6:** The moderating effect test of environmental cognition and social network: the CMP method.

Variable	Sample of Environmental Cognition on Local Off-Farm Employment	Sample of Environmental Cognition on Urban Off-Farm Employment	Sample of Social Network on Local Off-Farm Employment	Sample of Social Network on Urban Off-Farm Employment
	(7)	(8)	(9)	(10)
Household non-agricultural employment ratio	0.021	−3.601 ***	−4.009 ***	−3.535 ***
	(2.908)	(0.180)	(0.197)	(0.172)
Environmental cognition	0.101	−0.189		
	(0.135)	(0.079)		
Environmental cognition × Household non-farm employment ratio	0.015	1.056 **		
	(0.485)	(0.229)		
Social network			0.087 ***	−0.064 *
			(0.023)	(0.036)
Social network × Household non-farm employment ratio			0.365 ***	0.234 ***
			(0.065)	(0.079)
Control variable	YES	YES	YES	YES
Observations	704	353	704	353
Wald chi2	80.84 ***	1033.21 ***	1990.84 ***	963.25 ***

Note: ***, **, and * denote significant at 1%, 5%, and 10% level, respectively; robust standard errors are presented in parentheses; limited by space, the regression results of the control variables are not reported.

**Table 7:** Non-agricultural employment and domestic sewage discharge behavior of farmers: robustness test.

Variable	(11)	(12)	(13)
Household non-agricultural employment ratio		0.353 ***	2.566 ***
		(0.125)	(0.587)
Average non-agricultural employment hours	−0.334 ***		
	(0.088)		
Atanhrho_12	0.024 ***		−0.516 ***
	(0.042)		(0.162)
**First stage estimate**			
Village non-agricultural employment ratio	0.798 ***		0.573 ***
	(0.073)		(0.066)
Control variable	YES	YES	YES
Observations	2473	2473	1917
Wald chi2	914.48 ***	66.61 ***	357.78 ***

Note: *** denote significant at 1% level, respectively; robust standard errors are presented in parentheses; limited by space, the regression results of the control variables are not reported.

## Data Availability

The data underlying the results presented in the study are all available. The data presented in this study are available on request from the corresponding author. The data are not publicly available due to privacy.
